# The neural correlates of subjectively perceived and passively matched loudness perception in auditory phantom perception

**DOI:** 10.1002/brb3.331

**Published:** 2015-03-19

**Authors:** Dirk De Ridder, Marco Congedo, Sven Vanneste

**Affiliations:** 1 Department of Surgical Sciences, Section of Neurosurgery, Dunedin School of Medicine, University of OtagoDunedin, New Zealand; 2 BRAI²N, Sint Augustinus HospitalAntwerp, Belgium; 3 Vision and Brain Signal processing (ViBS) Research Group, GIPSA-Lab, CNRS, Grenoble UniversityGrenoble, France; 4 School of Behavioral and Brain Sciences, The University of Texas at DallasDallas, Texas

**Keywords:** Context, gamma, intensity, loudness, nesting, parahippocampus, theta, tinnitus

## Abstract

**Introduction:**

A fundamental question in phantom perception is determining whether the brain creates a network that represents the sound intensity of the auditory phantom as measured by tinnitus matching (in dB), or whether the phantom perception is actually only a representation of the subjectively perceived loudness.

**Methods:**

In tinnitus patients, tinnitus loudness was tested in two ways, by a numeric rating scale for subjectively perceived loudness and a more objective tinnitus-matching test, albeit it is still a subjective measure.

**Results:**

Passively matched tinnitus does not correlate with subjective numeric rating scale, and has no electrophysiological correlates. Subjective loudness, in a whole-brain analysis, is correlated with activity in the left anterior insula (alpha), the rostral/dorsal anterior cingulate cortex (beta), and the left parahippocampus (gamma). A ROI analysis finds correlations with the auditory cortex (high beta and gamma) as well. The theta band links gamma band activity in the auditory cortex and parahippocampus via theta–gamma nesting.

**Conclusions:**

Apparently the brain generates a network that represents subjectively perceived tinnitus loudness only, which is context dependent. The subjective loudness network consists of the anterior cingulate/insula, the parahippocampus, and the auditory cortex. The gamma band activity in the parahippocampus and the auditory cortex is functionally linked via theta–gamma nested lagged phase synchronization.

## Introduction

Nonpulsatile tinnitus is the percept of a sound that is not present in the environment, and can thus be considered an auditory phantom percept, which is analogous to phantom pain in the somatosensory system (De Ridder et al. 2011a). The most common cause of tinnitus is auditory deafferentation, which can result in behavioral changes such as hearing loss, but not all auditory deafferentation is associated with audiometric changes (Weisz et al. 2006). Partial cochlear nerve sections without audiometric changes is evidence that auditory deafferentation can exist without hearing loss (Dandy 1941). Recently, it has been proposed that tinnitus arises as a way to reduce environmental sensory uncertainty resultant from auditory deafferentation (De Ridder et al. 2014a). This is based on the free-energy principle, which has been proposed as a universal principle governing adaptive brain function and structure (Friston 2010), stating that the brain must minimize its free energy (i.e., must reduce by the process of perception its uncertainty (its prediction errors) about the environment). Brain mechanisms have evolved to fill in the missing information and therefore tinnitus might represent a filling in missing mechanism (De Ridder et al. 2014a), as most patients perceive the tinnitus in the deafferented frequencies (Norena et al. 2002), and initially attribute it as coming from the environment. This could suggest that principles of normal sensory perception might also apply to auditory phantom perception.

Perception is the act of interpreting and organizing a sensory stimulus to produce a meaningful experience of the world and of oneself (De Ridder et al. 2011a) in order to decrease the inherent uncertainty in the intra- and extrapersonal environment (De Ridder et al. 2014a). A stimulus produces an effect on the different sensory receptors inducing sensation. Further processing of this sensory stimulation generates an internal representation of the outer and inner world called a percept (De Ridder et al. 2011a). Thus, whereas sensation is related to ‘detecting stimuli’ in the environment, perception is related to ‘transforming stimuli into useful information’ of the environment or self.

Our understanding of sensation and perception is more limited in the case of phantom perception (De Ridder et al. 2011a), which is the conscious awareness of a percept in the absence of an external stimulus (Jastreboff 1990). A fundamental aspect of auditory phantom perception is how the individual consciously perceives his/her tinnitus. Does the brain create a network that represents the sound, as measured objectively? Or, does the brain only create a network that is a representation of the (subjective) phantom percept? In order to answer this simple yet fundamentally important question, the neural basis for determining whether phantom precept is a result of an objective or subjective sound representation is analyzed by source localized EEG activity and functional connectivity. This functional connectivity is further analyzed by looking at theta–gamma nesting which has been proposed as a mechanism governing communication between anatomically distant brain areas (Buzsaki and Draguhn 2004; Canolty et al. 2006; Lisman and Jensen 2013).

## Materials and Methods

### Participants

One-hundred and thirty-six patients (*M *=* *45.75 years; SD = 15.92; 58 males and 78 females) with chronic tinnitus were included in a study performed at the multidisciplinary TRI (Tinnitus Research Initiative) Clinic at the University Hospital Antwerp, Belgium after exclusion of individuals with pulsatile tinnitus, Ménière disease, otosclerosis, chronic headache, neurological disorders (i.e., brain tumors), and individuals being treated for mental disorders in order to increase the sample homogeneity. All patients were interviewed as to the perceived location of the tinnitus (the left ear, the right ear, in both ears, and centralized in the middle of the head as well the tone – pure tone like or noise-like). All patients were screened for the severity of hearing loss using the British Society of Audiology pure tone audiometry procedures (Electronics Orbiter 922 Version 2 in a soundproof audiometric booth using TDH-39 headphone as transducer) at 0.125 kHz, 0.25 kHz, 0.5 kHz, 1 kHz, 2 kHz, 3 kHz, 4 kHz, 6 kHz, and 8 kHz (Audiology 2008). Tinnitus loudness was tested in two ways: a numeric rating scale between 0 and 10 was used to evaluate the subjectively perceived tinnitus loudness based on the question: ‘How loud is your tinnitus?’ (0 = no tinnitus and 10 = as loud as imaginable’). A more objective measurement of the tinnitus loudness was performed using a tinnitus-matching test. In unilateral tinnitus patients, the tinnitus matching was performed contralateral to the tinnitus ear. In bilateral tinnitus patients, tinnitus matching was performed contralateral to the worst tinnitus ear. The tinnitus matching consisted of the assessment of the tinnitus pitch and loudness. First, a 1 kHz pure tone was presented contralateral to the (worst) tinnitus ear at 10 dB above the patient's hearing threshold in that ear. If the patient said the tinnitus tone sounded higher in pitch, the next presented tone would be an octave higher. If the patient said the tinnitus tone was lower, the, next tone would be an octave lower. Once the frequency region was established, half octave steps are used (up to 12 kHz). The pitch was adjusted until the patient judged the presented sound to resemble his/her tinnitus. The loudness of this tone was subsequently adjusted in a similar way until the contralaterally presented sound corresponded exactly to the patient's tinnitus, both in pitch and loudness. The level was increased 2 dB, if the patient said that their tinnitus was softer, and vice versa (up to 80 dB HL). The objective tinnitus loudness in decibels sensation level (dB SL) was computed by subtracting the presented sound intensity level in decibels hearing level (dB HL) with the auditory threshold at that frequency, in order to compensate for the hearing loss at the tinnitus frequency.

This study was approved by the local ethical committee (Antwerp University Hospital) and was in accordance with the declaration of Helsinki.

### Data collection

EEG data were obtained as a standard procedure. Recordings were obtained in a fully lighted room with each participant sitting upright on a small but comfortable chair. The actual recording lasted approximately 5 min. The EEG was sampled using Mitsar-201 amplifiers (NovaTech http://www.novatecheeg.com/) with 19 electrodes placed according to the standard 10–20 International placement (Fp1, Fp2, F7, F3, Fz, F4, F8, T7, C3, Cz, C4, T8, P7, P3, Pz, P4, P8, O1, O2), analogous to what is was done in the normative group. Impedances were checked to remain below 5 kΩ. Data were collected while the patient's eyes were closed (sampling rate = 500 Hz, band passed 0.15–200 Hz). Off-line data was band-pass filtered in the range 2–44 Hz, resampled to 128 Hz, and subsequently transposed into Eureka! Software (Congedo 2002). The data were then plotted and carefully inspected for manual artifact rejection. All episodic artifacts including eye blinks, eye movements, teeth clenching, body movement, or ECG artifact were removed from the stream of the EEG. Average Fourier cross-spectral matrices were computed for frequency bands delta (2–3.5 Hz), theta (4–7.5 Hz), alpha (8–12 Hz), low beta (13–21 Hz), high beta (21.5–30 Hz), and gamma (30.5–44 Hz).

### Source localization

Standardized low-resolution brain electromagnetic tomography (sLORETA; Pascual-Marqui 2002) was used to estimate the intracerebral electrical sources that generated the recorded activity (at sensory level). As a standard procedure, a common average reference transformation (Pascual-Marqui 2002) is performed before applying the sLORETA algorithm. sLORETA computes electric neuronal activity as current density (A/m^2^) without assuming a predefined number of active sources. The solution space used in this study and associated leadfield matrix are those implemented in the LORETA-Key software (freely available at http://www.uzh.ch/keyinst/loreta.htm). This software implements revisited realistic electrode coordinates (Jurcak et al. 2007) and the lead field produced by Fuchs et al. (2002) applying the boundary element method on the MNI-152 (Montreal neurological institute, Canada) template of Mazziotta et al. (2001). The sLORETA-key anatomical template divides and labels the neocortical (including hippocampus and anterior cingulated cortex) MNI-152 volume in 6239 voxels of dimension 5 mm^3^, based on probabilities returned by the Demon Atlas (Lancaster et al. 2000). The coregistration makes use of the correct translation from the MNI-152 space into the Talairach and Tournoux space (Brett et al. 2002).

sLORETA has received considerable validation from studies combining sLORETA with other more established localization methods, such as functional magnetic resonance imaging (fMRI) (Vitacco et al. 2002; Mulert et al. 2004), structural MRI (Worrell et al. 2000), positron emission tomography (PET) (Dierks et al. 2000; Pizzagalli et al. 2004; Zumsteg et al. 2005), and was used in previous studies to detect, for example, activity in the auditory cortex (van der Loo et al. 2009). Further sLORETA validation has been based on accepting as ground truth the localization findings obtained from invasive, implanted depth electrodes, in which case there are several studies in epilepsy (Zumsteg et al. 2006a,b,c) and cognitive ERPs (Volpe et al. 2007). It is worth emphasizing that certain deep structures such as the anterior cingulate cortex (Pizzagalli et al. 2001) and mesial temporal lobes (Zumsteg et al. 2006a,b,c) can be correctly localized with these methods.

Statistical analysis is based on estimating, via randomization, the empirical probability distribution for the max-statistic, under the null hypothesis comparisons (Nichols and Holmes 2002). This methodology corrects for multiple testing (i.e., for the collection of tests performed for all voxels, and for all frequency bands). Due to the nonparametric nature of the method, its validity does not rely on any assumption of Gaussianity (Nichols and Holmes 2002). sLORETA statistical contrast maps were calculated through multiple voxel-by-voxel comparisons in a logarithm of F-ratio (Pascual-Marqui 2002, 2007a,b; Pascual-Marqui et al. 2002). The significance threshold is based on a permutation test with 5000 permutations (Pascual-Marqui 2002, 2007a,b; Pascual-Marqui et al. 2002).

### Region of interest analysis

The log-transformed electric current density was averaged across all voxels belonging to the regions of interest for the different frequency bands delta (2–3.5 Hz), theta (4–7.5 Hz), alpha (8–12 Hz), low beta (13–21 Hz), high beta (21.5–30 Hz), and gamma (30.5–44 Hz). The regions of interest are the left and right primary (BA41) and secondary (BA21) auditory cortex.

A multivariate ANOVA (i.e., Wilks’ Lambda) for the frequency bands was used for the different frequency bands with the respective region of interest (i.e., left and right primary auditory cortex (BA41)), and left and right secondary auditory cortex (BA21) as dependent variables while using objective/subjective loudness as independent variables.

### Lagged Phase connectivity

Brain connectivity can refer to a pattern of anatomical links (“anatomical connectivity”), of statistical dependencies (“functional connectivity”) or of causal interactions (“effective connectivity”) between distinct units within a nervous system. Present research focuses on functional connectivity which captures deviations from statistical independence between distributed and often spatially remote neuronal units. Statistical dependence may be estimated by measuring correlation versus covariance, and spectral coherence versus phase locking. Functional connectivity is often calculated between all elements of a system, regardless of whether these elements are connected by direct structural links. Unlike structural connectivity, functional connectivity is highly time dependent. Statistical patterns between neuronal elements fluctuate on multiple time scales, some are as short as tens or hundreds of milliseconds. It should be noted that functional connectivity does not make any explicit reference to specific directional effects or to an underlying structural model.

Coherence and phase synchronization between time series corresponding to different spatial locations are usually interpreted as indicators of the “connectivity”. However, any measure of dependence is highly contaminated with an instantaneous, nonphysiological contribution due to volume conduction (Pascual-Marqui 2007a,b). However, Pascual-Marqui (2007a,b) introduced new measures of coherence and phase synchronization taking into accounts only noninstantaneous (lagged) connectivity, effectively removing the confounding factor of volume conduction. As such, this measure of dependence can be applied to any number of brain areas jointly, (i.e., distributed cortical networks, whose activity can be estimated with sLORETA). Measures of linear dependence (coherence) between the multivariate time series are defined. The measures are nonnegative, and take the value zero only when there is independence and are defined in the frequency domain: delta (2–3.5 Hz), theta (4–7.5 Hz), alpha (8–12 Hz), low beta (13–21 Hz), high beta (21.5–30 Hz), and gamma (30.5–44 Hz). Based on this principle lagged linear connectivity was calculated. Time series of current density were extracted for different region of interests using sLORETA. Power in all 6239 voxels was normalized to a power of 1 and log transformed at each time point. Region-of-interest values thus reflect the log-transformed fraction of total power across all voxels, and separately for specific frequencies. Regions of interest were defined based upon all brain areas obtained in previous analyses for the different frequency.

Connectivity contrast maps were calculated through multiple comparisons using t-statistics. The significance threshold was based on a permutation test with 5000 permutations. Again a comparison was made between the tinnitus group (recent onset and chronic tinnitus group) and the control subjects as well as between chronic tinnitus patients and tinnitus patients with recent onset.

### Theta–gamma nesting

It has been proposed that theta–gamma coupling (e.g., by nesting) is an effective way of communication between cortically distant areas (Canolty et al. 2006). To verify whether this theta–gamma nesting is present in passively matched or subjective tinnitus perception, theta–gamma nesting is calculated between those areas that are functionally connected by theta lagged phase synchronization. Theta–gamma nesting was computed as follows: first, the time series for the *x*, *y*, and *z* component of the sLORETA current for each ROI was obtained. Next, it was filtered in the theta (4–7.5 Hz) and gamma (30.5–44 Hz) frequency band-pass regions. Those are the time series of the current in the three orthogonal directions in space. In each frequency band and for each ROI, a principal component analysis was computed and the first component was retained for theta and gamma. The Hilbert transform was then computed on the gamma component and the signal envelope retained. Finally, the Pearson correlation between the theta component and the gamma envelope was computed. With this procedure, each correlation was computed on one-second of data and a correlation was available for each sample, from which the percent time for which the correlation is above a threshold (theta–gamma nesting) can be computed.

The amount of theta–gamma nesting was defined as the percentage of time a correlation was obtained of 0.15 or higher. We opted to select 0.15 as this is the cut-off to obtain a *P-*value of 0.05 based on the rationale that at least 3 min of uncontaminated EEG generates 180 epochs. If *n* = 180, 0.15 is *r*-score to obtain significance. For each individual, we calculated the percentage of time (how many epochs of theta–gamma nesting had a correlation higher than 0.15) there was a theta–gamma nesting for the left parahippocampal area and the left secondary auditory cortex. These percentages of time were then correlated with both the objective and subjective measure of loudness.

## Results

### Behavioral measurements

**Figure 1:**
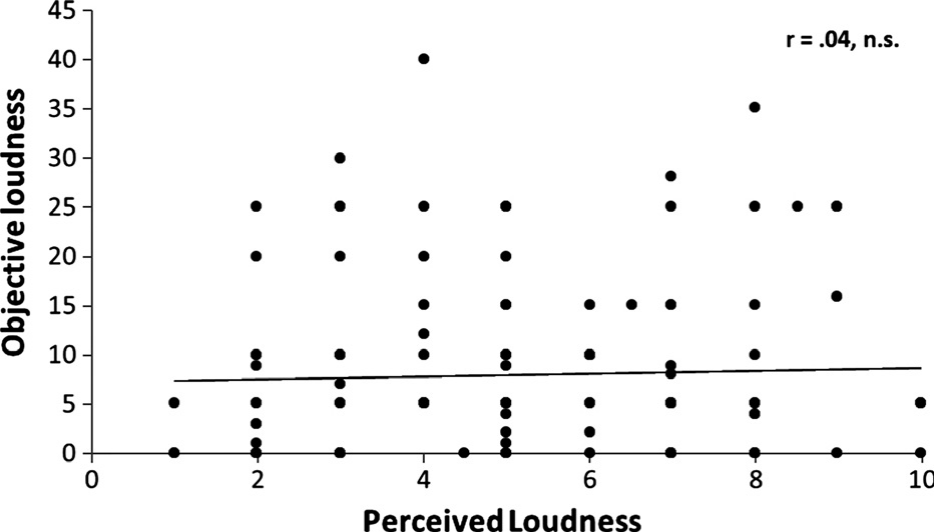
A Pearson correlation between the passively matched loudness and the subjectively perceived loudness reveals no significant effect.

A Pearson correlation revealed no significant association between the more objective loudness as measured with tinnitus matching (dB SL) and the subjectively perceived loudness as measured with NRS loudness (*r* = −0.04, n.s.) (see Fig.1).

### Whole-brain analysis

**Figure 2:**
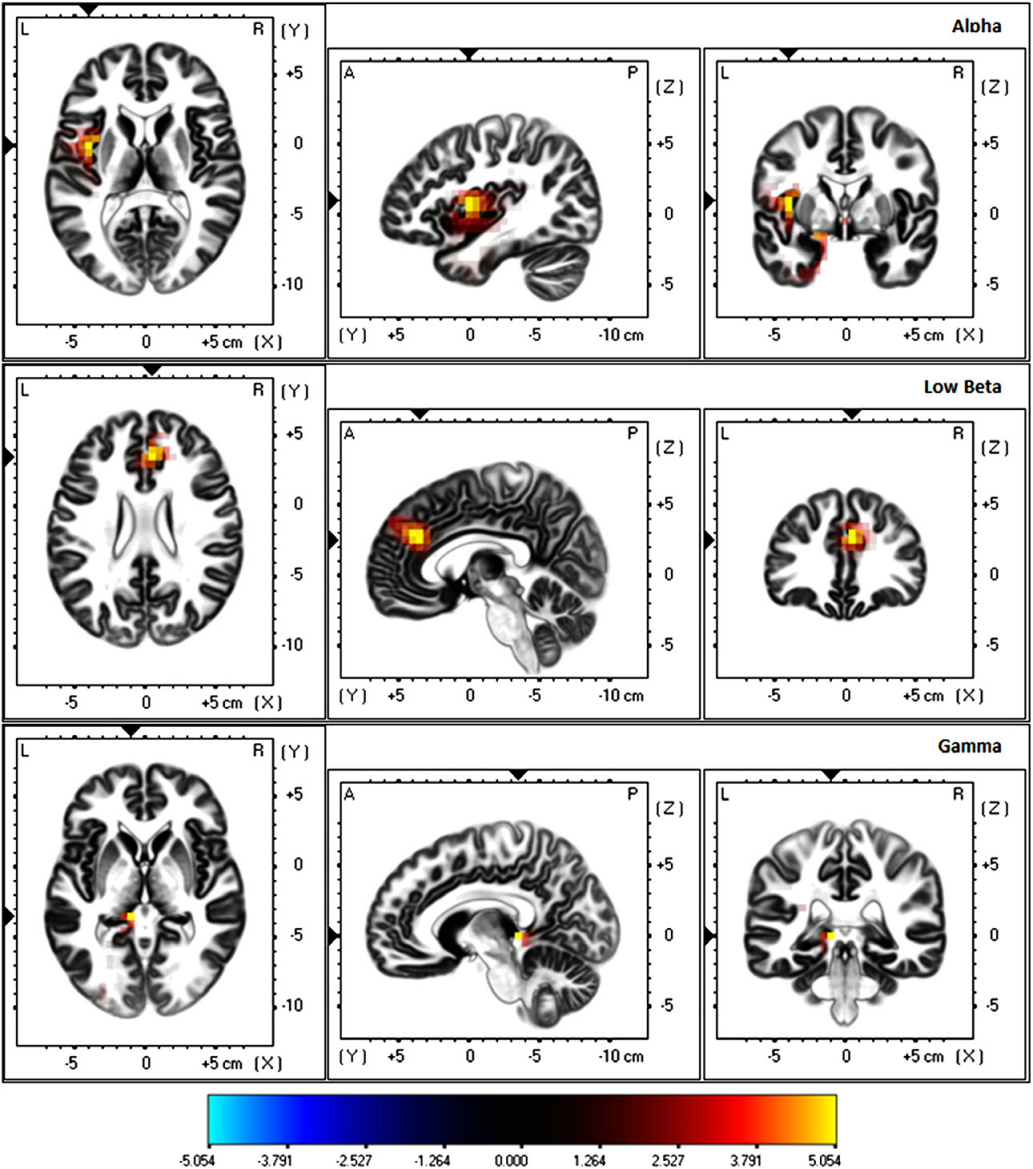
Significant positive correlations between the subjectively perceived tinnitus loudness on a numeric rating scale and brain activity in the alpha (left insula), low beta (right dorsal anterior cingulate cortex), and gamma (left parahippocampus) frequency band.

Correlation analysis between the subjectively perceived loudness on a NRS and source localized current density brain activity revealed a significant positive correlation (*P *<* *0.05) with the left insula for alpha band activity, with an area located between the pregenual and dorsal anterior cingulate cortex for low beta activity as well as with the left parahippocampus for the gamma frequency band (see Fig.2). Therefore, the higher the subjective loudness on NRS, the higher activity is within the left anterior insula, the rostral/dorsal anterior cingulate cortex, and the left parahippocampus. No significant correlations were obtained for delta, theta, and high beta.

Correlation analysis between the passively matched tinnitus loudness and brain activity revealed no significant correlations for the delta, theta, alpha, low beta, high beta, nor gamma frequency bands.

### Region-of-interest analysis

A MANOVA including subjectively perceived loudness (NRS) and passively matched tinnitus loudness as independent variables and the different log-transformed current densities for the frequency bands, namely, delta (2–3.5 Hz), theta (4–7.5 Hz), alpha (8–12 Hz), low beta (13–21 Hz), high beta (21.5–30 Hz), and gamma (30.5–44 Hz), at the left primary auditory cortex as dependent variable revealed a significant effect for subjective loudness in the high beta (*F *=* *4.25, *P *<* *0.05, *β *= 0.16) and gamma band (*F *=* *6.70, *P *<* *0.05, *β *= 0.17), but not for delta, theta, alpha, and low beta. Therefore, an increase in subjectively perceived loudness is associated with an increased current density in the high beta and gamma frequency bands in the left primary auditory cortex. No significant effect could be found for passively matched tinnitus loudness.

**Figure 3:**
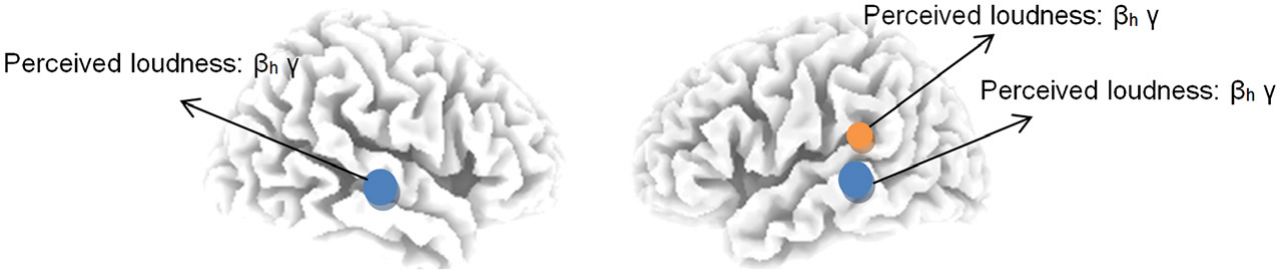
The log-transformed current density shows significant correlations between the high beta and gamma activity and subjective tinnitus loudness perception in the left primary (yellow) and bilateral secondary (blue) auditory cortex on a region-of-interest analysis.

A similar analysis was conducted for the left secondary auditory cortex. This revealed again a significant effect for subjective loudness (NRS) for the log-transformed current density for high beta (*F *=* *7.08, *P *<* *0.01, *β *= 0.16) and gamma band (*F *=* *5.01, *P *<* *0.05, *β *= 0.16), but not for delta, theta, alpha, or low beta. Thus, an increase in the subjectively perceived loudness (NRS) is associated with an increased current density in the high beta and gamma frequency band in the left secondary auditory cortex (Fig.3).

Again, no significant effect could be obtained for passively matched tinnitus loudness.

For the right secondary auditory cortex, a significant effect could be demonstrated for subjective loudness and the log-transformed current density for high beta (*F *=* *5.36, *P *<* *0.19) and gamma (*F *=* *4.21, *P *<* *0.05, *β *= 0.18), but not for delta, theta, alpha, and low beta. These effects suggest that an increase in the subjectively perceived loudness is associated with increased current density in the high beta and gamma frequency band in the right secondary auditory cortex. No significant effect could be obtained for passively matched loudness.

For the right primary auditory cortex, no significant effects could be obtained in both the subjectively perceived loudness (NRS) as well as the passively matched loudness for delta, theta, alpha, low beta, high beta, and gamma (summary of results in figure 3).

### Lagged phase connectivity

**Figure 4:**
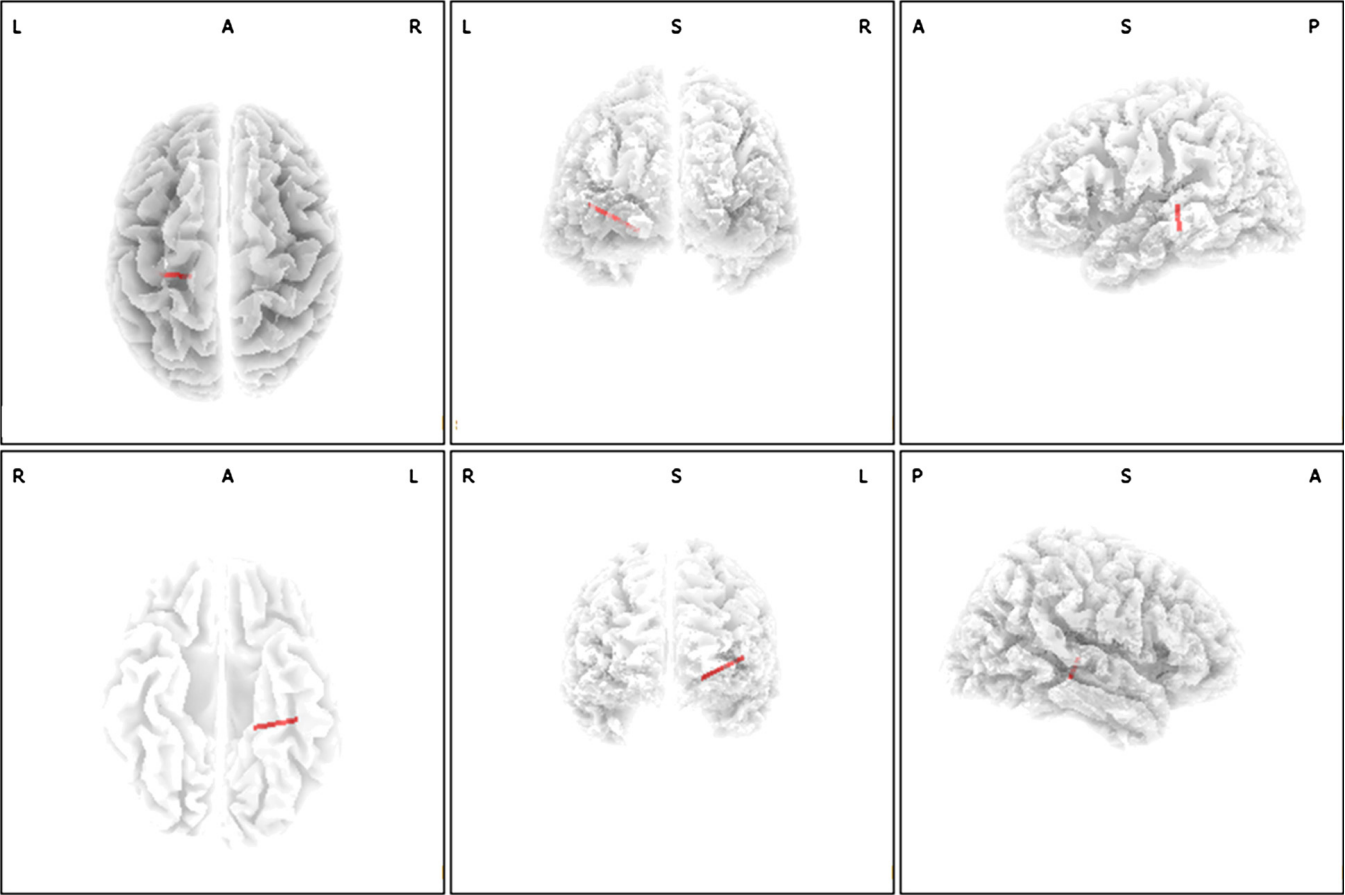
A significant correlation between the subjective loudness and lagged phased connectivity between the left parahippocampus and the left secondary auditory cortex for the theta frequency band (*r* = 0.45, *P *<* *0.05).

A correlation was obtained between the subjectively perceived loudness and lagged phased connectivity between the left parahippocampus and the left secondary auditory cortex for the theta frequency band (*r* = 0.45, *P *<* *0.05) (see Fig.4). This suggests that a stronger lagged phase connectivity between left parahippocampus and the left secondary auditory cortex is associated with the subjective loudness or vice versa. No significant effect was obtained for delta, alpha, low beta, high beta, and gamma.

A similar correlation analysis was also conducted for the passively matched tinnitus loudness and lagged phased connectivity. However, this analysis revealed no significant effect for, respectively, the delta, theta, alpha, low beta, high beta, and gamma.

### Theta–gamma nesting

**Figure 5:**
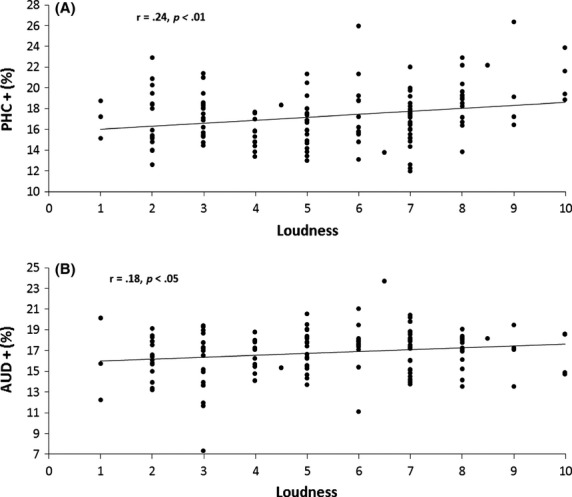
(A) The percentage of time theta–gamma nesting is present is positively correlated with the subjectively perceived loudness for the left parahippocampus. (B) A positive correlation is noted between the percentage of time there is theta–gamma nesting and the perceived loudness for the left secondary auditory cortex.

This analysis revealed that the percentage of time there was a theta–gamma nesting in the left parahippocampus is positively correlated with the subjectively perceived loudness (*r* = 0.24, *P *<* *0.01, Fig.5A), but not with the passively matched tinnitus loudness (*r* = −0.06, n.s). This finding indicates that the more theta–gamma nesting there is in the left parahippocampus the louder the tinnitus perceived by the patients.

A positive correlation was also obtained between the percentage of time there was a theta–gamma nesting in the left secondary auditory cortex and the perceived loudness (*r* = 0.18, *P *<* *0.05, Fig.5B), but not with the passively matched tinnitus loudness (*r* = 0.08, n.s), suggesting that an increased percentage of theta–gamma nesting is associated with subjectively louder tinnitus perception.

## Discussion

This simple but fundamental study evaluates the difference between the neural basis of a subjective loudness report, ‘how loud is your tinnitus?’ and a more objective report of the loudness, obtained by tinnitus matching, as a simplified version of the question: does the brain store objective representations or is subjectivity intrinsically embedded in the network encoding the phantom percept?

Two fundamentally different possibilities exist. (1) The brain holds a neural representation of the objective loudness and links this to a nonspecific emotional state such as the distress level (Vanneste et al. 2010; De Ridder et al. 2011c, 2014a) or (2) the subjectively perceived loudness is an emergent property from a stored electrophysiological network encompassing both auditory and affective brain areas.

The study has five interrelated important findings. The first important finding is that there is no correlation between the objectively measured loudness and the subjectively perceived loudness. A second finding is that the brain has a neural representation for the subjective loudness percept, but not for the more objective loudness percept. A third finding is that the subjectively perceived loudness is related to high beta and gamma band activity in the auditory cortex, which replicates previous results. A fourth finding is that the subjectively perceived loudness is determined by the functional connectivity in the theta band between the auditory cortex and an auditory memory-related area (parahippocampus) and is lateralized to the left. Finally, the communication between the parahippocampal area and the auditory cortex involves theta–gamma nesting, (i.e., the gamma activity in the auditory cortex that correlates with subjective loudness perception is determined by the phase of the theta activity), which functionally connects the auditory cortex to the parahippocampal area.

### No correlation between the objective and the perceived loudness

It is interesting that there is no correlation between the perceived loudness of the phantom sound and the more objectively measured matched loudness. This is in agreement with other studies previously published (Burns 1984; Hallam et al. 1985; Moller 1994; Savastano 2004). This suggests that they represent something fundamentally different. Conceptually, this is understandable as the subjectively perceived tinnitus loudness is very context dependent: The subjectively perceived loudness in a silent environment is very different than in a noisy environment where the tinnitus might be masked. The passively matched tinnitus loudness is always determined in a soundproof booth.

### The brain has a neural representation for the subjective loudness percept, but not for the objective loudness

Ecologically it makes sense that there is no neural template for the passively matched tinnitus loudness. Storing an objective loudness electrophysiologically as a representation in the brain might indeed be useless, as loudness is ecologically always dependent on the noise in the environment (a 50 dB sound at the roaring seaside is very different than the same 50 dB in a soundproof room). Furthermore, hearing acuity levels change in individuals, for example, in presbyacusis, clearly limiting the value of storing representations of objective loudness. This is in agreement with fMRI studies that demonstrate that the BOLD activation in the auditory cortex is more closely related to the subjective percept of a stimulus (i.e., loudness) rather than to its physical characteristics (i.e., intensity) (Hall et al. 2001; Langers et al. 2007). Therefore, phantom sound (tinnitus) perception seems to behave electrophysiologically in a similar way as perception of externally presented tones.

### Neural correlates of subjectively perceived tinnitus loudness

The neural correlates of subjective tinnitus loudness perception involve a coactivation of the anterior cingulate cortex, the insula, and the parahippocampal area. A further ROI analysis supports the involvement of the auditory cortex as well. The auditory cortex involvement in tinnitus loudness has been demonstrated before. Tinnitus perception has been correlated with sustained high-frequency gamma band activity in temporal areas in humans in quantitative electroencephalographic (QEEG) (Ashton et al. 2007) and magnetoencephalographic studies (MEG) (Llinás et al. 1999; Llinas et al. 2005; Weisz et al. 2005, 2007). There is a sound-level-dependent activation of the primary auditory cortex in humans as investigated with EEG and fMRI (Lenz et al. 1998; Mulert et al. 2005), with an increasing primary auditory cortex activation for increasing loudness, and there is an analogous tinnitus sound-level-dependent high beta and gamma band activation in the auditory cortex (van der Loo et al. 2009). In this study, this correlation is confirmed both for high beta/gamma band activity, in the left primary and bilateral secondary auditory cortex.

The involvement of the rostral/dorsal anterior cingulate cortex and insula is unsurprising as well. The combined activity of the anterior cingulate cortex and insula reflects salience (Seeley et al. 2007) (i.e., the behavioral relevance of stimuli) (Fecteau and Munoz 2006) for predicting future outcomes (Behrens et al. 2007), essential in a Bayesian brain model for tinnitus (De Ridder et al. 2014a). This combined rostral/dorsal ACC and insula activity in tinnitus is related to distress (Vanneste et al. 2010; De Ridder et al. 2011c; van der Loo et al. 2011), and has been attributed to a persisting (Strand et al. 2011) salience attribution to an otherwise unimportant sound (De Ridder et al. 2011a, 2014a). This fits with physiological sound perception, in which ongoing activity fluctuations in the anterior cingulate and insula determine whether a near threshold stimulus is detected or not (Sadaghiani et al. 2009). In tinnitus, the perceived loudness is also correlated with activity in the rostral/dorsal anterior cingulate cortex, insula cortex, auditory cortex, and is identical to real sound stimuli. This might be the reason why an auditory phantom percept is initially almost always attributed by the patient to an external sound source. This is because when people try to remember a sound, (i.e., when it is internally attributed), the dorsal anterior cingulate cortex and insula are deactivated (Rinne et al. 2009).

The parahippocampal involvement in the same gamma band range as the auditory cortex is also of interest. The posterior parahippocampal area has a sensory gating function for irrelevant or redundant auditory input (Boutros et al. 2008). The parahippocampal area has been hypothesized to play a central role in memory recollection, sending information from the hippocampus to the association areas, and a dysfunction in this mechanism is posited as an explanation for complex auditory phantom percepts such as auditory hallucinations (Diederen et al. 2010). As the parahippocampal area is involved in tinnitus and tinnitus distress (Vanneste et al. 2010), a similar mechanism has been proposed for tinnitus (De Ridder et al. 2011a, 2014a).

It has been shown that the functional connectivity between the parahippocampus and the subgenual anterior cingulate is involved in tinnitus distress (Vanneste et al. 2010; De Ridder et al. 2011c; Joos et al. 2012). In a recent study, it was shown that a very selective and frequency specific 10 and 11.5 Hz functional connection, as measured by lagged phase synchronization between the parahippocampus and subgenual anterior cingulate cortex, determines whether or not a person is severely (10 Hz) or very severely (11.5 Hz) distressed by his/her tinnitus (Vanneste et al. 2014). The connectivity between the parahippocampal area and subgenual anterior cingulate cortex/ventromedial prefrontal cortex is likely a part of a general aversive network involving the cerebellum, parahippocampal area, and hypothalamus. These are all activated both by pain and unpleasant visual images (Moulton et al. 2011). Unfortunately, EEG cannot pick-up electrical activity from the cerebellum or the hypothalamus.

In view of these functional connections, it is important to see whether a similar mechanism might be involved in tinnitus loudness perception.

### Loudness-related functional connectivity between parahippocampal area and auditory cortex

How loud the tinnitus is subjectively perceived is related to the theta lagged phase synchronization between the left parahippocampal area and left auditory cortex. This suggests that how loud a phantom sound is perceived might actually be determined by the distress that the brain attaches to the phantom sound or the opposite, as functional connectivity does not look at directionality. Therefore, it is also possible that gamma activity in the auditory cortex correlates with the perceived loudness and influences the distress the brain attaches to the phantom sound.

According to these analyses, it becomes clear that the parahippocampal area is a critical component in tinnitus. Its functional connectivity to the subgenual ACC in alpha oscillations determines how much distress and depression a patient feels in association with the phantom sound (Vanneste et al. 2010; De Ridder et al. 2011c; Joos et al. 2012). Its functional connectivity in theta oscillations to the auditory cortex determines how loud the sound is perceived. The parahippocampal involvement might therefore be related to the contextual influences that determine how loud and how stressful the tinnitus is perceived. This is in line with what has recently been proposed to be a major function for the parahippocampus, namely, contextually influencing perception (Aminoff et al. 2013). This functional connectivity between the left parahippocampal area and auditory cortex has already been shown, using resting state fMRI, to be an essential neurophysiological feature of tinnitus (Maudoux et al. 2012). We now propose that this functional connectivity is clinically linked to the subjectively perceived loudness, and explain the underlying mechanism involved: theta–gamma nesting.

### Gamma band activity nested on theta functional connectivity links auditory cortex to parahippocampus

Communication between the parahippocampal area and the auditory cortex involves theta–gamma nesting, that is, the gamma activity in the auditory cortex that correlates with subjective loudness perception is determined by the phase of the theta activity, which functionally connects the auditory cortex to the parahippocampal area. This is in accordance with a general concept of theta–gamma nesting (Buzsaki and Draguhn 2004; Canolty et al. 2006; Lisman and Jensen 2013). It has been demonstrated that higher frequency gamma oscillations are confined to a small neuronal space, whereas very large networks are recruited during slow oscillations (von Stein and Sarnthein 2000). This permits for theta activity to synchronize large spatial domains and to bind together specific assemblies of anatomically restricted higher frequency oscillations by the appropriate timing (Buzsaki and Chrobak 1995; Engel et al. 2001; Varela et al. 2001). In a recent case report with electrodes implanted on the auditory cortex for tinnitus suppression, it was demonstrated that theta–gamma coupling is increased when the patient perceives tinnitus in comparison with when he or she perceives no tinnitus (De Ridder et al. 2011b). This suggests that the theta activity might be the carrier wave required for coactivation of the tinnitus loudness network (Schlee et al. 2008, 2009) and that gamma activity encodes the subjectively perceived tinnitus loudness (van der Loo et al. 2009), which is consistent with the data of this study. Furthermore, in physiological auditory perception, gamma nesting on theta waves in a distributed network of brain areas is involved in control of auditory attention (Doesburg et al. 2012).

## Conclusion

This study shows that the brain encodes the subjective loudness of a phantom sound, but not the passively matched tinnitus loudness. There is no correlation between the objectively measured loudness and the subjectively perceived loudness. The subjectively perceived loudness is related to the amount of distress the person feels in contrast to the passively matched loudness. For the passively matched loudness, no cerebral correlates can be found. The subjectively perceived loudness is encoded by activity in multiple areas, consisting of the rostral/dorsal anterior cingulate, insula and parahippocampus, as well as the auditory cortex. How loud a phantom sound is perceived critically depends on the lagged phase theta functional connectivity between the parahippocampal area and auditory cortex, and the loudness encoding gamma oscillations in the auditory cortex are functionally linked to the parahippocampal area via nesting on the theta wave.
